# Targeted Delivery of Liposomal Temozolomide Enhanced Anti-Glioblastoma Efficacy through Ultrasound-Mediated Blood–Brain Barrier Opening

**DOI:** 10.3390/pharmaceutics13081270

**Published:** 2021-08-17

**Authors:** Zhuqing Song, Xiuxian Huang, Jieqiong Wang, Feiyan Cai, Ping Zhao, Fei Yan

**Affiliations:** 1 Department of Breast Surgery, Peking University Shenzhen Hospital, Shenzhen 518036, China; songzhuqing@163.com; 2 Paul C. Lauterbur Research Center for Biomedical Imaging, Institute of Biomedical and Health Engineering, Shenzhen Institutes of Advanced Technology, Chinese Academy of Sciences, Shenzhen 518055, China; xiuxian_huang@163.com (X.H.); fy.cai@siat.ac.cn (F.C.); 3 Center for Cell and Gene Circuit Design, CAS Key Laboratory of Quantitative Engineering Biology, Shenzhen Institute of Synthetic Biology, Shenzhen Institutes of Advanced Technology, Chinese Academy of Sciences, Shenzhen 518055, China; jq.wang@siat.ac.cn; 4 Department of Ultrasound, Guangzhou University of Traditional Chinese Medicine First Affiliated Hospital, Guangzhou 510405, China

**Keywords:** glioblastoma, blood–brain barrier, ultrasound, temozolomide, liposome

## Abstract

Glioblastoma (GBM) is the commonest form of primary brain tumor in the central nervous system, with median survival below 15 months and only a 25% two-year survival rate for patients. One of the major clinical challenges in treating GBM is the presence of the blood–brain barrier (BBB), which greatly limits the availability of therapeutic drugs to the tumor. Ultrasound-mediated BBB opening provides a promising approach to help deliver drugs to brain tumors. The use of temozolomide (TMZ) in the clinical treatment of GBM has been shown to be able to increase survival in patients with GBM, but this improvement is still trivial. In this study, we developed a liposomal temozolomide formulation (TMZ-lipo) and locally delivered these nanoparticles into GBM through ultrasound-mediated BBB opening technology, significantly suppressing tumor growth and prolonging tumor-bearing animal survival. No significant side effects were observed in comparison with control rats. Our study provides a novel strategy to improve the efficacy of TMZ against GBM.

## 1. Introduction

Glioblastoma (GBM) is the most common form of primary brain tumor in the central nervous system, with a median survival below 15 months and only a 25% two-year survival rate [1,2]. Although many treatment options, including surgical resection, radiotherapy, and chemotherapy, are currently available, they only marginally improve the survival time of patients and have little benefit on tumor recurrence [3,4,5]. Complete resection of GBM is virtually impossible due to its heterogeneous and infiltrative nature, and tumor relapse is almost inevitable [6]. Survival advantages have been demonstrated with postoperative radiation therapy at doses of 5000–6000 cGy, but this treatment has been shown to increase toxicity without additional survival benefit beyond 6000 cGy. In the past decades, pharmacological progresses have been made, leading to the development of molecular targeted therapies or precision medicine approaches [7]. Various novel drug formulations, delivery systems, and tumor-targeting strategies to inhibit the tumor progression or metastasis of GBM have been being widely studied [8,9,10]. However, the blood–brain barrier (BBB) is still a major limitation reducing the efficacy of anti-cancer drugs in the treatment of GBM patients [11,12]. 

Temozolomide (TMZ), a monofunctional DNA alkylating agent, has been used for the treatment of newly diagnosed GBM, but the effect of TMZ is highly schedule dependent [13]. Genetic or acquired resistances to TMZ are easily developed, and a strict regimen must be followed in order to obtain a favorable result [14,15]. Researchers have started to uncover mechanisms that underlie TMZ resistance in GBM, finding that the enzyme O6-methylguanine-DNA methyltransferase (MGMT) may remove methyl groups from DNA and repair the TMZ-induced DNA damage through DNA mismatch repair cascades [16,17,18]. Various TMZ combination treatment strategies with the RNAi silencing of MGMT or small-molecule inhibitors have been developed [19,20]. Unfortunately, TMZ must be administered at high doses in order to achieve therapeutic effects because of its short plasma half-life (only 1.8 h). This drug often induces a series of side effects, such as bone marrow depression, nausea, vomiting, headache, etc. [21]. Research works have demonstrated that the encapsulation of TMZ in nanoparticles such as liposomes or polymer nanoconjugates could enhance its anti-cancer efficacy and reduce toxicity to some degree in animal models of GBM [22,23,24]. Although GBM is well known to compromise the structural integrity of the BBB, causing some nanoparticles to be leaky at the tumor core, the BBB surrounding the proliferating cells at the tumor’s edge remains intact [11]. Therefore, in order to improve the efficacy of TMZ therapy in GBM, it is necessary to develop nanotechnology-based systems able to penetrate the BBB in order to target drug delivery to maximize the drug concentration at the brain tumor and to reduce related toxicity and side effects.

Focused ultrasound (US) combined with microbubbles (MBs) has been shown to be able to transiently and reversibly open the BBB without damaging neural cells [25,26]. Low-frequency focused ultrasound can pass through the skull and induce MBs in the blood to generate a series of cavitation effects including acoustic radiation force, microstreaming, microjet, etc., which leads to the destruction of the BBB. The presence of MBs may greatly decrease the US energy, making drug delivery into the brain safer. Given that the BBB is a lipophilic membrane structure located near to the microvascular endothelial network within the capillaries of the central nervous system (CNS) [27], in this study we encapsulated TMZ into liposomes and applied focused US to locally deliver them into the GBM.

## 2. Materials and Methods

### 2.1. Materials

1,2-Dipalmitoyl-sn-glycero-3-phosphocholine (DPPC) and cholesterol were purchased from Avanti Polar Lipids Inc. (Alabaster, AL, USA). TMZ, ICG, DMSO, sodium fluorescein and 4′,6-diamidino-2-phenyl indole (DAPI) were obtained from Sigma-Aldrich (St. Louis, MO, USA). Dulbecco’s modified Eagle’s medium (DMEM) was obtained from GIBCO (Grand Island, NY, USA). Fetal bovine serum (FBS) and 1% penicillin/streptomycin were from Invitrogen (Thermo Fisher Scientific, Inc., Waltham, MA, USA). Cell Counting Kit-8 (CCK-8) was purchased from Dojindo Laboratories (Tokyo, Japan), the In Situ Cell Death Detection Kit (TUNEL) was purchased from Roche (Indianapolis, IN, USA). Anti-caspase-3 antibody and anti-ZO-1 antibody were obtained from eBioscience (Ireland, UK). bEnd.3 and C6 tumor cells were purchased from the American Type Culture Collection. Rats were obtained from Guangdong Medical Experimental Animal Center (Guangzhou, China). All other reagents were of analytical grade.

### 2.2. Preparation of TMZ-Lipo

The thin-film hydration method was used to fabricate the TMZ-lipo [28]. Firstly, TMZ was dissolved in dimethyl sulfoxide (DMSO) at a concentration of 19.4 mg/mL. An amount of 20 mg DPPC and 4.5 mg cholesterol were solved in chloroform at a 7:3 molar ratio. The thin lipid film was formed after the organic solvents were removed under a nitrogen flow, followed by further drying for over 3 h under vacuum. Then, the dried lipid film was hydrated with ultrasonic wave at 65 °C with 0.4 mL phosphate-buffered saline (PBS, pH 7.4) containing 100 μL of stoke TMZ solution (19.4 mg/mL) to obtain a final TMZ-lipo formulation. The resulting TMZ-lipo was extruded through a polycarbonate carbonate filter (0.1 μm, Avanti Polar Lipids, Alabaster, AL, USA). The free TMZ was separated from the TMZ-lipo by ultrafiltration (5000 Da molecular weight cutoff). Indocyanine-green-loaded liposomes (ICG-lipo) were prepared according to our previous report [29]. MBs were prepared in-house as described in our previous study [30].

### 2.3. Characterization of TMZ-Lipo

The average diameter, zeta potential, and size distribution of TMZ-lipo were analyzed by dynamic light scattering (Zetasizer Nano ZS, Malvern Instruments, Malvern, UK). A 1 mL TMZ-lipo sample (1:100 dilution) was used for the analysis. Transmission electron microscopy (TEM, JEM-2100F, JEOL, Tokyo, Japan) with negative staining was used for the morphological examination. Before analysis, 100 μL of TMZ-lipo (1:100 dilution) was placed on a carbon-coated copper grid and stained with 2% (*w*/*v*) phosphotungstic acid. After air-drying, they were observed by TEM. The TMZ-lipo was dissolved in DMSO/methanol (1:9, *v*/*v*) mixture and analyzed using a UV–visible spectrophotometer at 330 nm. The drug encapsulation efficiency (EE) was determined by the equation below. EE (%) = Drug entrapped × 100/Total amount of drug.

### 2.4. In Vitro Release Assay

The in vitro drug release was studied using a dialysis technique. In brief, 3 mL of TMZ-lipo suspension (concentration 1 mg/mL) was loaded in a dialysis bag (molecular weight cutoff 8–14 kDa) and dialyzed against 100 mL of PBS with pH 6.5 or pH 7.4 at 37 ± 0.5 °C in a water bath shaker at 100 rpm. The dishes were closed to prevent evaporation of the release medium. At regular time intervals, 0.2 mL of the release medium was collected and then the same volume of fresh release medium was added. The amount of TMZ in the medium was measured by UV absorbance [31].

### 2.5. In Vitro BBB Model and US Irradiation

The in vitro BBB experimental system was setup for US sonication. Briefly, bEnd.3 and C6 tumor cells were used to develop the in vitro BBB model. The abluminal side of the membrane was first treated with poly-l-lysine (100 μg/mL), and then 1 × 10^5^ bEnd.3 cells were seeded on the luminal side of Transwell system (12 mm diameter, with 0.4 μm pores, Corning Incorporated Life Sciences, Tewksbury, MA, USA) and allowed to grow for 5 days in a CO_2_ incubator at 37 °C to form a cell layer. After 6 days, 5 × 10^5^ C6 cells were seeded at the abluminal side of the membrane and further cultivated for 24 h. The permeability of the in vitro BBB model to sodium fluorescein was measured by adding 100 μg/mL sodium fluorescein to the upper chamber in serum-free medium. At regular time intervals, 0.2 mL of the release medium was collected from the lower chamber and replaced with the same volume of fresh release medium. The fluorescence intensity of sodium fluorescein in the lower chamber was determined at 485 nm excitation and 528 nm emission wavelength. The permeability coefficient Papp (cm/s) was calculated based on Fick’s law, according to a previous report [32]. A 1 MHz focused US transducer was driven by a function generator and amplified by a power amplifier for transmission of treatment pulses. The apparatus consisted of a transducer and a removable water cone. The water cone was used to direct the ultrasound beam into the cell culture dish. A US-transparent polyurethane membrane was placed between the cell culture dishes. US sonication (1 MHz) was applied at 0.2 MPa acoustic pressure for 10,000 cycles, with 1 Hz PRF and a sonication duration of 1 min.

### 2.6. Permeability Assay of TMZ-Lipo

Delivery of TMZ or TMZ-lipo (before or after US) across cell monolayers was quantified to evaluate their permeability. After treatment with 38.8 μg/mL TMZ solution or TMZ-lipo containing equivalent TMZ, US irradiation was applied. At regular time intervals, 0.1 mL of the release medium was collected and then the same volume fresh release medium was added. The amount of TMZ in the medium was measured by HPLC as described earlier [33]. In brief, a C18 column was used for analyses under UV detection at 330 nm at 35 °C. Methanol/acetic acid 0.5% (30:70, *v*/*v*) was used as the mobile phase, eluting at 1.1 mL/min isocratic flow rate.

### 2.7. Cell Viability Assay

C6 cells were cultured in high-glucose DMEM containing 10% fetal bovine serum (FBS), 100 U/mL penicillin, and 100 μg/mL streptomycin at 37 °C. The C6 cells were seeded into a 96-well microplate at a density of 10^5^ cells/well. At 60–70% confluence, the cells were treated with the indicated concentrations of the TMZ or TMZ-lipo at concentrations of 0.97 μg/mL, 1.94 μg/mL, or 19.4 μg/mL for 12 or 24 h prior to the measurement of the cell viability. Cell viability was evaluated by Cell Counting Kit-8 (CCK-8). The percentage of viable cells was obtained by the following formula: Cell viability rate (%) = OD_treated group_/OD_control group_ × 100.

### 2.8. Cellular Immunofluorescence

The bEnd.3 cells were washed with PBS and then fixed with 4% paraformaldehyde, followed by permeabilization with 0.3% Triton X-100. BSA (1%) was used for blocking for 1 h. Then, the cells were stained successively with rabbit anti-mouse ZO-1 primary antibody and Alexa-Fluoro_488_-labeled goat anti-rabbit secondary antibody. After staining with DAPI, these cells were examined by a confocal laser scanning microscope (TCS SP5, Leica, Wetzlar, Germany).

### 2.9. In Vivo Drug Distribution

GBM-bearing Sprague-Dawley rats (200–250 g) were used for the biodistribution studies. The animals were fasted 12 h before drug administration. The free TMZ or TMZ-lipo solution was injected through the tail vein at a TMZ dosage of 20 mg/kg, and the TMZ-lipo + US group was treated with US. Following drug administration, three rats in each group were then sacrificed at 2 h. The spleen, kidneys, liver, brain (tumor), heart, and lungs were surgically collected and washed with PBS. Each organ sample was washed with PBS. After weighting samples to approximately 100 mg, tissue samples were homogenized with 1 mL PBS by homogenizer. Tissue homogenates were centrifugated, and the supernatant was passed through a 0.22 μm filter. Approximately 20 μL of the filtered sample was used for HPLC analysis.

### 2.10. Tumor Model

Male Sprague-Dawley rats (200–250 g) were used to develop the brain tumor model. Animals received care in accordance with the guidelines for the care and use of laboratory animals. All animal experiments were approved by Shenzhen Institute of Advanced Technology, Chinese Academy of Sciences Animal Care and Use Committee (Ethical approval No. SIAT-IRB-180208-YGS-YF-A0442, approved on 15 March 2018). Rats were anesthetized with 3% isoflurane gas and immobilized on a stereotactic frame (RWD, Shenzhen). A hole in the skull was made by a small dental drill in order to expose the injection site (1 mm anterior and 3 mm lateral to the bregma). C6 tumor cells were harvested and resuspended (5 × 10^5^/10 μL) in DMEM for implantation into the striatum of rat brains. Ten microliters of C6 cell suspension were injected at a depth of 5 mm from the brain surface to build the tumor model.

### 2.11. In Vivo Anti-Tumor Study

The growth of tumor in the rat brain was monitored by MRI using turbo-spin-echo-based T2-weighted images 10 days following tumor cell implantation. Animals were divided into 5 sub-groups: (1) the control (intravenous administration of 1 mL of PBS, n = 6); (2) the MBs + US group, administered only MBs and US irradiation (n = 6); (3) the TMZ group, 20 mg/kg TMZ injection through tail vein each day for 3 days (n = 6); (4) the TMZ-lipo group, 20 mg/kg TMZ-lipo injection through tail vein each day for 3 days (n = 6); (5) the TMZ-lipo group, 20 mg/kg TMZ-lipo injection in tail vein plus US-BBB opening each day for 3 days (n = 6). The injected volume was 1 mL for each rat. The US parameters used to open the BBB were as follows: transducer central frequency = 1 MHz; acoustic pressure = 800 kPa; pulse repetition frequency = 1 Hz; cycle number = 10,000; exposure time = 1 min; MB dosage = 5 × 10^8^ bubbles; exposure time = 1 min.

### 2.12. Histological Analysis

After the rats were sacrificed, their major organs, including spleen, lung, heart, liver, kidney, and brain (tumor), were harvested and fixed with formalin, followed by embedding in paraffin. Tissue samples were cut in 7 μm sections by a paraffin slicing machine. H&E staining was used to examine the tissue damage and histological changes. To quantitatively assess apoptosis, terminal deoxynucleotidyl transferase-mediated dUTP nick-end labeling (TUNEL) was carried out according to the manufacturer’s protocol. As for immunohistochemical staining of caspase-3, the cells or tumor sections were washed and immersed in 4% paraformaldehyde at room temperature for 10 min. Endogenous peroxidase was inactivated by incubation with 3% H_2_O_2_ in methanol for 10 min at room temperature. After that, the sections were incubated with rabbit polyclonal anti-caspase-3 antibody (diluted 1:200) at 4 °C overnight. The slides were photographed and measured using a computer-assisted image analysis system (NIH ImageJ 1.57 software, National Institutes of Health, Tokyo, Japan).

### 2.13. Statistical Analysis

All values shown are expressed as mean ± SD. Statistical analysis was performed by 22.0 SPSS software (SPSS, Chicago, IL, USA) and ANOVA was used to compare the differences between groups. Significant or very significant differences were considered at the levels *p* < 0.05 or *p* < 0.01 for these groups, respectively.

## 3. Results

### 3.1. Preparation and Characterization of TMZ-Lipo

**Figure 1:**
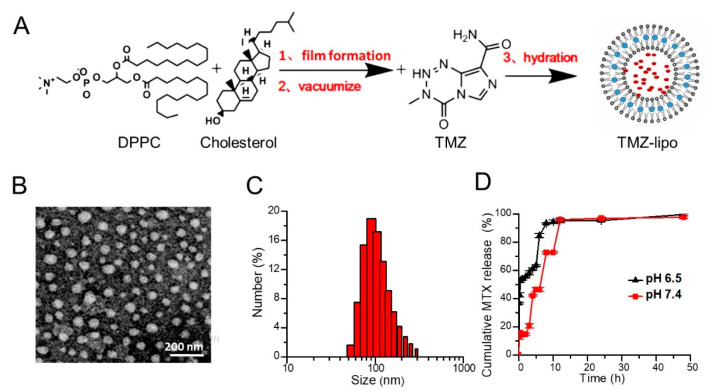
Fabrication and characterization of the TMZ-lipo. (**A**) Schematic diagram of the fabrication of TMZ-lipo. (**B**) Representative micrograph obtained by transmission electron microscopy. Scale bar = 200 nm. (**C**) Particle size distribution of the TMZ-lipo obtained by dynamic light scattering. (**D**) In vitro drug release profile of TMZ-lipo in PBS at pH 6.5 and pH 7.4 (*n* = 3).

TMZ-lipo was prepared by thin-film rehydration followed by extrusion, as shown in Figure 1A. The TEM image shows that the TMZ-lipo had a spherical structure, with a relatively uniform size distribution in the range 50–100 nm (Figure 1B). Dynamic light scattering (DLS) showed an average hydrodynamic radius of 148.13 ± 2.66 nm, with a polydispersity index (PDI) of 0.23 ± 0.05, indicating a suitable particle size and distribution (Figure 1C) for targeted drug delivery. The zeta potential of the TMZ-lipo was found to be −23.14 ± 0.50 mV. The encapsulation efficiency achieved 52% for TMZ-lipo. The release behavior of the TMZ-lipo solution in vitro is shown in Figure 1D. The results show that TMZ could be released from TMZ-lipo at both pH 7.4 and 6.5, but the release rate was faster in the acidic pH 6.5 environment than at physiological pH 7.4 in the first 10 h. This could be attributed to the fact that TMZ-lipo was more stable at pH 7.4. After 10 h, there were no obvious differences in the release rates of the drug from the liposomes, because most drugs had been released by this time.

### 3.2. In Vitro Cytotoxic Effects of TMZ-Lipo on C6 Cells

**Figure 2:**
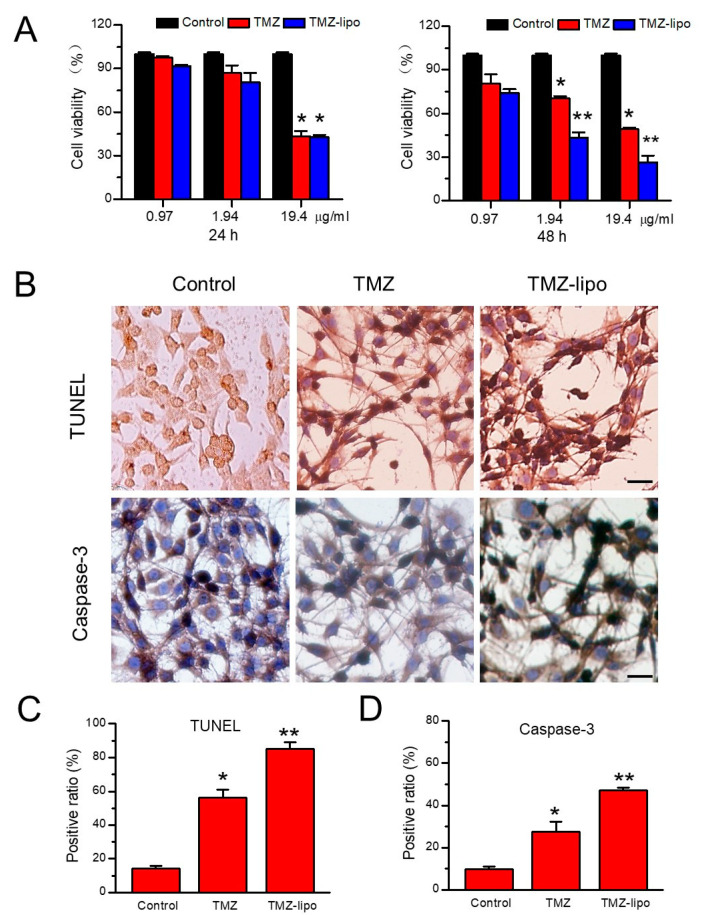
In vitro assay of TMZ-lipo. (**A**) CCK-8 assay showing that TMZ-lipo decreased C6 cell viability after 24 and 48 h. (**B**) Cell apoptosis assay by TUNEL and immunohistochemical staining of caspase-3 for cells treated with TMZ and TMZ-lipo after 48 h. Scale bar, 10 μm, * *p* < 0.05, ** *p* < 0.01. (**C**) Percentage of TUNEL-positive cells. (**D**) Percentage of caspase-3-positive cells.

In order to determine the cytotoxic effects of TMZ-lipo on the C6 tumor cells, free TMZ or TMZ-lipo at a concentration of 0.97, 1.94, or 19.4 µg/mL was added to the cell suspensions, and the cell viabilities were detected by CCK-8 assay after 24 or 48 h. Figure 2A shows that both the free TMZ and TMZ-lipo had a comparable cytotoxicity in C6 cells after 24 h. It is notable that TMZ-lipo induced significantly stronger cell death than free TMZ at the concentrations of 1.94 µg/mL and 19.4 µg/mL after 48 h (Figure 2A). The better tumor cell-killing effects of TMZ-lipo over free TMZ has been demonstrated in a previous document [23]. TUNEL and caspase-3 staining assays further demonstrated that the TMZ-lipo induced more C6 cell apoptosis after 48 h, as shown in Figure 2B. Quantitative analysis revealed that there were 54.34 ± 3.67% TUNEL-positive cells and 47.04 ± 1.58% caspase-3-positive cells for TMZ-lipo versus 14.28 ± 1.57 % TUNEL-positive cells and 27.42 ± 4.85% caspase-3-positive cells for free TMZ (Figure 2C,D).

### 3.3. In Vitro BBB Permeability after US Irradiation

**Figure 3:**
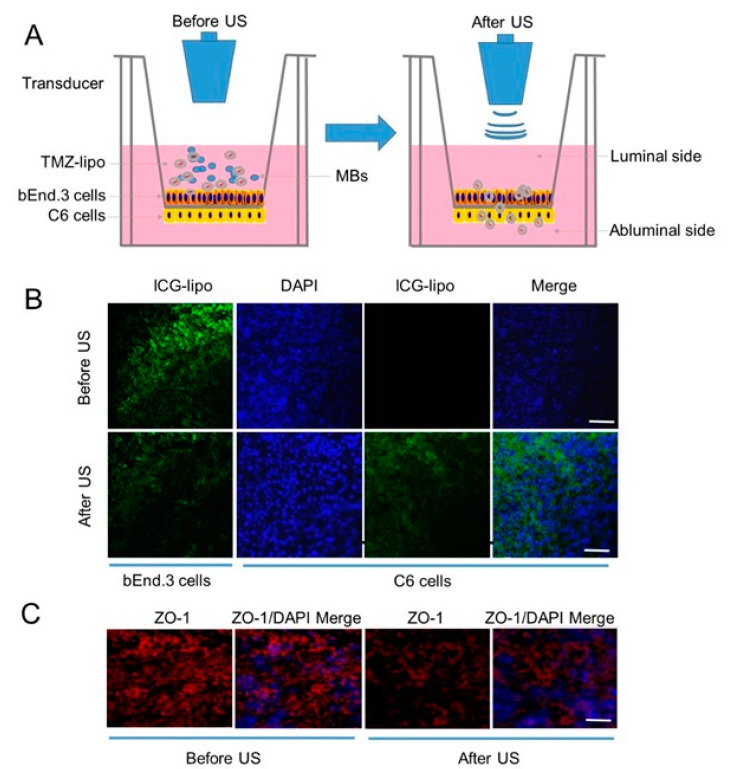
BBB permeability after US in the in vitro BBB model. (**A**) Schematic diagram of the in vitro BBB cell model and US-mediated drug delivery across the BBB. (**B**) The confocal fluorescence microscopic images show fluorescein sodium across the in vitro BBB cell model. Scale bar = 20 µm. (**C**) The level of TJ-associated protein ZO-1 was detected by immunohistochemical staining. Scale bar = 20 µm.

In order to analyze whether US combined with MBs promoted the permeability of drugs across the BBB, an established in vitro BBB model was used for this work (Figure 3A). Indocyanine-green-loaded liposomes (ICG-lipo) were used as the model drug for convenience of observation. Figure 3B shows the confocal fluorescence microscopic images of ICG-lipo traveling from the luminal side of the bEnd.3 cells to the abluminal side of the C6 cells before or 2 h after US irradiation. Only bEnd.3 cells showed fluorescence, and no signals were shown in the C6 cells before US irradiation; this is because ICG-lipo did not permeate across the barrier of bEnd.3 cells to enter ethe C6 cells. However, 2 h after US irradiation, fluorescence was observed in the C6 cells on the abluminal side, and the fluorescence from the bEnd.3 cells on luminal side had decayed significantly (Figure 3B). This result indicates that ICG-lipo had permeated across the barrier of bEnd.3 cells and entered the C6 cells upon receiving US irradiation. To investigate the TJ integrity of the bEnd.3 cells, the TJ-associated protein ZO-1, which links cell adhesion molecules to the actin cytoskeleton, was immunoassayed. The ZO-1 of bEnd.3 cells in the BBB model showed continuous, smooth fluorescent signals and was mainly restricted to cell–cell junctions before US. However, ZO-1 expression was apparently decreased after US irradiation (Figure 3C). Given the fact that the US opened the in vitro BBB from the tight junction protein ZO-1, it would improve the permeability to drugs.

### 3.4. In Vitro Cellular Toxicity

**Figure 4:**
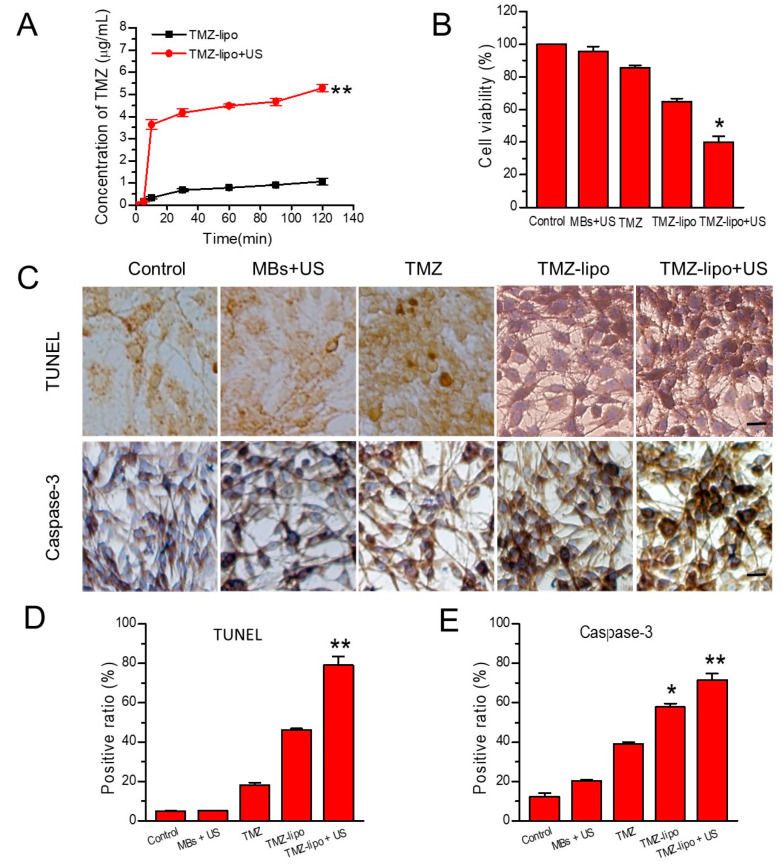
Cytotoxic effects of TMZ-lipo on C6 cells in the BBB model. (**A**) TMZ concentrations across the in vitro BBB model treated with TMZ-lipo, or TMZ-lipo + US. (**B**) Viability of cells treated with MB, TMZ, TMZ-lipo, or TMZ-lipo + US. (**C**) Representative images of TUNEL and immunohistochemical staining after treatment in the in vitro BBB model. Scale bar = 10 μm; * *p* < 0.05, ** *p* < 0.01. (**D**) Percentage of TUNEL-positive cells. (**E**) Percentage of caspase-3-positive cells.

Significantly enhanced permeation efficiency was also found in the in vitro cell BBB model when using TMZ-lipo + US irradiation (Figure 4A). Five groups were included to test the cytotoxic effects of TMZ-lipo in the C6 cells seeded on the abluminal side of the in vitro BBB model: control, MBs + US, free TMZ, TMZ-lipo, and TMZ-lipo + US. As shown by CCK-8 assay, the control and MBs + US groups did not influence the C6 viability. However, the TMZ-lipo + US group had significantly reduced C6 cell viability compared with TMZ-lipo and free TMZ groups after 48 h at 38.8 μg/mL (*p* < 0.05) (Figure 4B). To further examine the level of cell apoptosis, TUNEL and caspase-3 immunohistochemical staining assays were utilized, and the results are shown in Figure 4C. Figure 4C demonstrates that the control and MBs + US groups did not influence the C6 viability, but TMZ-lipo + US treatment significantly increased the TUNEL-positive and caspase-3-positive cell ratios in comparison with the other groups. Similarly, the TMZ-lipo + US group also had significantly reduced C6 cell viability compared with the TMZ-lipo and free TMZ groups after 48 h at 38.8 μg/mL. Quantitative analysis revealed there were 78.98 ± 4.53% TUNEL-positive cells and 71.41 ± 3.37% caspase-3-positive cells for TMZ-lipo + US versus 46.24 ± 0.69% and 17.98 ± 1.52% TUNEL-positive cells for TMZ-lipo and free TMZ, and 57.73 ± 1.86% and 39.14 ± 0.83% caspase-3-positive cells for free TMZ, respectively.

### 3.5. TMZ Concentration Distribution

**Figure 5:**
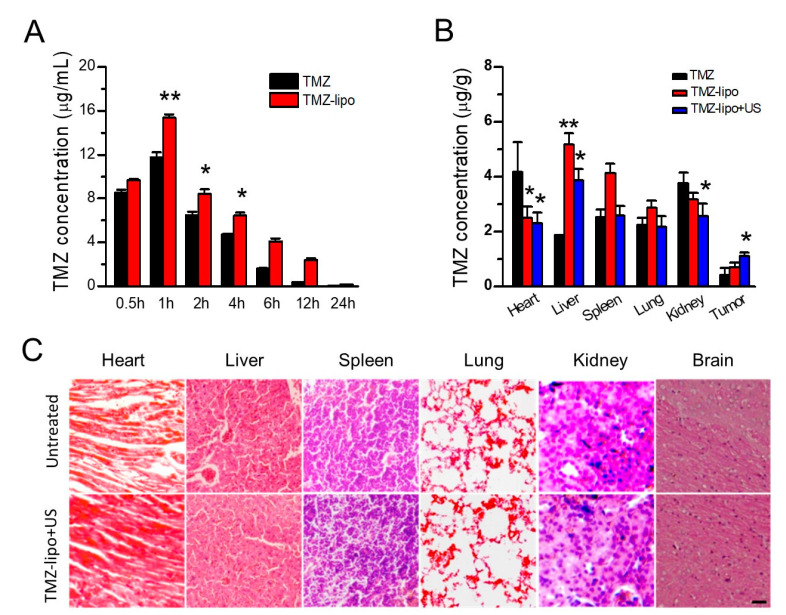
Biodistribution of the TMZ in the free TMZ, TMZ-lipo, and TMZ-lipo + US treatment groups. (**A**) TMZ concentrations in plasma after intravenous administration of TMZ or TMZ-lipo after 2 h. * *p* < 0.05, ** *p* < 0.01. (**B**) Biodistribution of TMZ in different tissues after intravenous injection of free TMZ, TMZ-lipo, or TMZ-lipo + US, 2 h after US irradiation. * *p* < 0.05, ** *p* < 0.01. (**C**) Representative histological images of heart, liver, spleen, lung, kidney, and brain stained with H&E after intravenous injection of TMZ-lipo after 24 h (Scale bar, 100 μm).

To detect the plasma TMZ concentrations in vivo, the SD rats were injected with free TMZ or TMZ-lipo at 20 mg/kg. Figure 5A shows the mean drug concentration in plasma versus time after administration of the TMZ or TMZ-lipo. Obviously, the plasma TMZ concentration from the TMZ-lipo group was much higher than that of the free TMZ group after 1 h, achieving the peak concentration of 15.36 ± 0.30 μg/mL in comparison to 11.74 ± 0.46 μg/mL for the free TMZ group. The TMZ-lipo concentration was 2.38 ± 0.15 μg/mL after 12 h while the free TMZ was almost undetectable, indicating that TMZ entrapped in liposomes would remain in the circulation for a longer time than the free TMZ. It might be explained by the protection of the lipid bilayer membranes, slowing drug release from liposomes [14]. In order to detect the biodistribution of TMZ after the intravenous injection of TMZ-lipo combined with US-mediated BBB opening, the brain tumor, liver, heart, kidney, spleen, and lung samples were collected for HPLC analysis after 2 h US irradiation. Figure 5B shows that TMZ-lipo combined with US induced an elevated TMZ-lipo level in brain tumor in comparison with the free TMZ or TMZ-lipo treatments, indicating that US-mediated BBB opening could enhance drug delivery into the brain tumor. Notably, the drug concentrations in heart, liver, and kidney tissues were significantly lower after treatment with the TMZ-lipo + US, compared with TMZ or TMZ-lipo treatments (*p* < 0.01). Next, histological analysis was performed using of H&E staining. No appreciable abnormalities were observed in the main organs in comparison with the untreated control organs, showing that TMZ-lipo + US was tolerable and had no obvious side effects (Figure 5C).

### 3.6. In Vivo Anti-Tumor Efficacy

**Figure 6:**
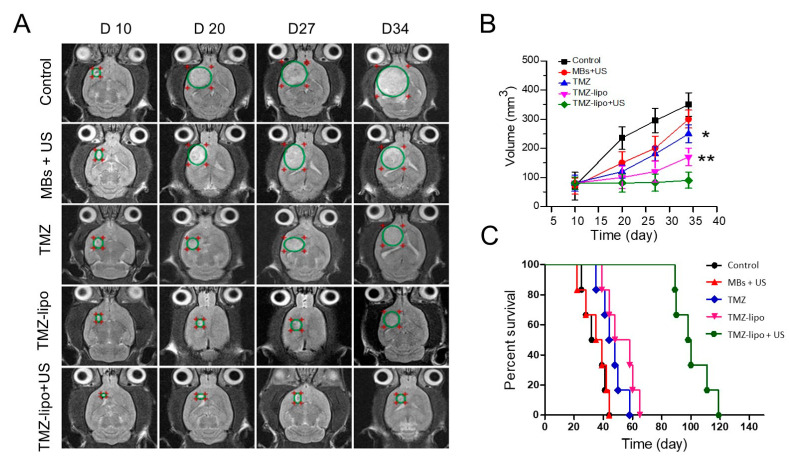
Therapeutic efficacy of TMZ-lipo in the GBM animal model. (**A**) Representative MR images to monitor brain tumor progression weekly from day 10 to day 34 following the completion of treatment. (**B**) Tumor growth curves from day 10 to day 34 in each group. * *p* < 0.05, ** *p* < 0.01. (**C**) Animal survival curves by Kaplan–Meier plot.

To determine the in vivo anti-tumor efficacy, an orthotopic transplanted C6 tumor model was developed. The tumor-bearing rats received treatments of PBS (control), MBs + US, free TMZ, TMZ-lipo, or TMZ-lipo + US. Typical T2-weighted MRI images in weekly follow-up to monitor brain tumor progression are shown in Figure 6A, and are quantified in Figure 6B. Tumors progressed rapidly in the control animal group. The MBs + US group did not show obvious tumor suppression effects, and was similar to the control group. The 20 mg/kg TMZ or TMZ-lipo administration partly inhibited tumor growth. By contrast, 20 mg/kg TMZ-lipo combined with US exposure provided nearly complete tumor progression suppression (*p* < 0.01). The survival curves from the Kaplan–Meier plot of the five groups are shown in Figure 6C, revealing that TMZ-lipo combined with US opening of the BBB significantly improved the percent survival when compared to control, free TMZ, or TMZ-lipo treatments (*p* < 0.01). These results indicate that TMZ-lipo had a stronger anti-tumor efficacy in the GBM animal model when combined with US-mediated BBB opening.

### 3.7. Histochemical Assay

**Figure 7:**
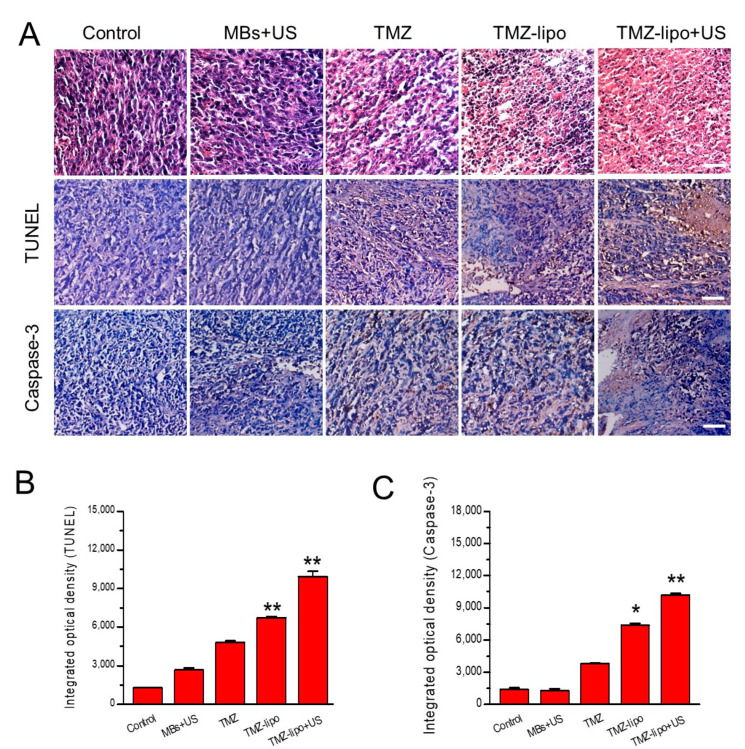
The detection of TUNEL and caspase-3. (**A**) H&E, TUNEL staining, and caspase-3 levels of brain tumors were analyzed. Scale bar = 100 μm. (**B**) Quantitative analysis of TUNEL-positivity levels by mean optical densities. (**C**) Quantitative analysis of caspase 3-positivity levels by mean optical densities. * *p* < 0.05, ** *p* < 0.01.

Finally, the apoptosis levels were examined using H&E, TUNEL, and caspase-3 immunohistochemical staining. Figure 7 shows that the control and MBs + US groups did not show severe structural damage or apoptotic cells. Only minor structural damage and few apoptotic cells could be found in the TMZ and TMZ-lipo groups. In contrast, significant tumor necrosis and more apoptotic cells could be observed in the tumors of the group receiving TMZ-lipo combined with US-mediated BBB opening versus the control group (Figure 7A,B). Similarly, significantly more caspase-3-positive cells could be observed in the TMZ-lipo + US group compared with control, MBs + US, free TMZ, or TMZ-lipo groups (Figure 7A,C).

## 4. Discussion

The most important function of the blood–brain barrier (BBB) is to prevent the brain from potential damage from harmful substances circulating in the blood, which is accomplished thanks to its unique barrier properties [34]. However, the potential pharmaceutical drugs to treat brain tumors have been severely limited by this barrier, bringing a huge challenge to translate them to clinical application. Given the fact that BBB remains relatively intact in aggressive brain tumors, especially in the tumor peripheral region, it is necessary to develop an approach to temporarily open the BBB to allow large-molecule drugs to penetrate brain tumors [35]. Focused US combined with MBs provides a promising method to locally and temporarily open the BBB [36]. A clinical trial has demonstrated that US in combination with systemic MBs injection is safe and well-tolerated in patients with recurrent GBM [25].

Although it can be administered orally and pass the BBB in clinical applications, TMZ has a half life of less than 2 h in the circulation and does not reach therapeutic concentrations at the tumor site. Liposomal carriers can modulate the biopharmaceutical properties of the encapsulated drugs [37]. Additionally, lipid DPPC is one of the most abundant components in the cell membranes of eukaryotic organisms. DPPC/cholesterol liposomes can maintain the stability of TMZ and extend its circulation time [38]. In our study, we used bEnd.3 cells to establish the in vitro BBB model, which is a cell line from brain-derived endothelial cells, with similar structural features to the BBB. In order to mimic the in vivo condition, we used bEnd.3 and C6 tumor cells to develop the in vitro BBB model. BBB opening by US combined with MBs promoted the delivery of TMZ-lipo into the brain tumor. More importantly, a locally enhanced drug delivery could be achieved because the BBB was only opened at the region irradiated by the acoustic beam. With the guidance of MRI, the acoustic beam from the transducer could be precisely directed on the brain tumor, achieving targeted delivery of TMZ-lipo for the treatment of glioma. As shown in Figure 5, compared with the brain which did not receive US irradiation, significantly enhanced TMZ drug concentration was observed in the brain tumor after US-mediated BBB opening treatment. It is also notable that significantly lower TMZ distribution appeared in the heart, liver, and kidney tissues in this treatment group in comparison with the groups administered TMZ with no US treatment, indicating that the side-effects from TMZ on these organs could be avoided or alleviated.

In this study, the enhanced therapeutic efficacy of TMZ-lipo when combined with US-mediated BBB opening was supported by our observation of tumor progression inhibition and increased survival. The enhanced therapeutic efficacy of TMZ-lipo may be attributed to greater drug accumulation in the tumor. It is also notable that we used MRI to monitor the progress of glioblastoma in our study. These MRI images provided us with a direct view of the tumor growth inhibition effect of TMZ-lipo combined with US-mediated BBB opening. Furthermore, we examined whether the TMZ-lipo could induce glioma cell apoptosis by using TUNEL assays and immunohistochemical staining of caspase-3. Our results revealed that the rates of apoptosis in US-treated tumors were significantly higher those in tumors not administered US treatment in the TUNEL assays. Significantly increased levels of caspase-3 in the tumors receiving TMZ-lipo + US may account for the higher cell apoptosis since caspase-3 is a critical marker of genotoxic-stress-induced apoptosis.

## 5. Conclusions

We successfully fabricated TMZ-lipo and examined its tumor growth inhibition effect when administered in combination with US-mediated BBB opening. The resulting TMZ-lipo had an average hydrodynamic particle size of 148.13 ± 2.66 nm. When combined with US irradiation, TMZ-lipo showed a significantly stronger C6 tumor-cell-killing activity in the in vitro BBB model. More effective delivery of TMZ-lipo across the BBB into the GBM could be observed in the mice receiving US-mediated BBB opening treatment, leading to significantly stronger tumor growth inhibition and longer animal survival. Histochemical staining assay confirmed there were more apoptotic tumor cells in the mice treated with TMZ-lipo + US, indicating the superior anti-tumor efficacy observed in this group. Our study suggests that US-mediated BBB opening is a promising technology for delivering TMZ-lipo into the brain to treat glioblastoma.

## Data Availability

Data is contained within the article.
